# Hard Times

**Published:** 1868-09

**Authors:** 


### HARD TIMES.

"Old Times" mournfully recede into the dim distance of the past, to memory growing dearer every hour, but "hard times" have been in the living present from our very earliest remembrance. In multitudes of places in New York at this moment, there are men who will tell you that they are barely earning a living ; not a few are falling behind hand, and are regretfully using the savings of previous years. The patent plan for killing off old "Hard Times," and he ought to have been dead long ago, is to live on less, in two directions.

Not long since a man told me that if he had life to live over again, and wanted to be sure of patronage and a fortune, he would go into the "Back and Belly Business ;" that was not only a new alliteration to us, but a new line of trade, and we asked what was its nature ; his reply was, that the people would always want clothes and something to eat, hence the butcher and the clothier would always have customers and cash customers too as long as there was a dime in circulation. As "dieting" is not another name for starvation in the teach.

ings of this Journal, but to eat a plenty of the good things of this life, well prepared; so in "living on less," as to dress, we do not advocate wearing old clothes or purchasing inferior materials, but to buy the best articles at the lowest prices, by patronizing honest men who have moderate ambitions and plain tastes, and who are consequently satisfied with very small profits, often turned. Fathers and mothers owe it to themselves, their children and to society, to hunt out these men. A. T. Stewart laid the foundation of his colossal fortune by rigidly adhering to the simple practice of selling his goods just for what they were. He charged the customary price, but our wives and daughters always felt an assurance that their purchases were all that they were represented to be and their confidence was gained and kept for life. A business man of our acquaintance* has taken a step in advance of the Napoleon of dry goods and not only guarantees that the goods he sells are all that they are represented to be, but that the price is lower than is asked for a similar quality of goods at any house of standing in all New York. The less it costs a man to live the less laborious will be his life, the longer will it last and the more genially and mellow will be his advancing years and eventually, without an ache or a pain, he will dry up entirely, will evaporate and leave this mundane sphere, without a jolt or a jar.

